# Supplementary material for: Gdf3 is required for robust Nodal signaling during germ layer formation and left-right patterning
Source: eLife. 2017 Nov 15;6:e28635. doi: 10.7554/eLife.28635 (PMC5745080; doi:10.7554/eLife.28635)
Supplement: Transparent reporting form [file elife-28635-transrepform.docx]

***eLife’s* transparent reporting form**

We encourage authors to provide detailed information *within their submission* to facilitate the interpretation and replication of experiments. If you have any questions, please contact us: [editorial@elifesciences.org](mailto:editorial@elifesciences.org).

**Sample-size estimation**

- You should state whether an appropriate sample size was computed when the study was being designed
- You should state the statistical method of sample size computation and any required assumptions
- If no explicit power analysis was used, you should describe how you decided what sample (replicate) size (number) to use

Please outline where this information can be found within the submission (e.g., page numbers or figure legends), or explain why this information doesn’t apply to your submission:

Sample size was chosen based on the internal variability of the assays and our experience with the early embryo (Goyal et al. Nature Genetics, 2017; PMID# 28166211). If an embryo was damaged during the process of staining and mounting, that embryo was excluded from the analysis.

**Replicates**

- You should report how often each experiment was performed
- You should include a definition of biological versus technical replication
- The data obtained should be provided and sufficient information should be provided to indicate the number of independent biological and/or technical replicates
- If you encountered any outliers, you should describe how these were handled
- Criteria for exclusion/inclusion of data should be clearly stated
- High-throughput sequence data should be uploaded before submission, with a private link for reviewers provided (these are available from both GEO and ArrayExpress)

Please outline where this information can be found within the submission (e.g., page numbers or figure legends), or explain why this information doesn’t apply to your submission:

The analysis of Bmp signaling and *eve1* ventral expansion in Figure 3 was performed once with multiple embryos (n values included in figure legend and source data files will be included with submission). If an embryo was damaged during the process of staining and mounting, that embryo was excluded from the analysis. Otherwise, all embryos were analyzed. The knockdown experimental results reported in Figure 4 are from single experiments utilizing the same clutch of embryos. However, these experiments have been performed multiple times, and yielded comparable results each time. The rescue experiments in Figure 5A were performed once for each combination of injection amount and mutant condition shown on the figure. The injections for each indicated mRNA injection amount were performed on different days and mutant embryo batches except for the 50 pg injections. These injections were performed on the same day for the MZ*gdf3* and M*gdf3* embryos using the same injection mixture. The same is true for Figures 5B and 5C, where for each figure independently, the injections of mRNA were performed on the same day with the same injection mixture into MZ*gdf3* and M*gdf3* embryos.

**Statistical reporting**

- Statistical analysis methods should be described and justified
- Raw data should be presented in figures whenever informative to do so (typically when N per group is less than 10)
- For each experiment, you should identify the statistical tests used, exact values of N, definitions of center, methods of multiple test correction, and dispersion and precision measures (e.g., mean, median, SD, SEM, confidence intervals; and, for the major substantive results, a measure of effect size (e.g., Pearson's r, Cohen's d)
- Report exact p-values wherever possible alongside the summary statistics and 95% confidence intervals. These should be reported for all key questions and not only when the p-value is less than 0.05.

Statistical test used for comparing data is applicable to Figure 3 and is included in the figure legend. Sample sizes are included in the figure legend or figure for Figure 3 and 4, respectively. Exact p-values were reported in Figure 3.

Please outline where this information can be found within the submission (e.g., page numbers or figure legends), or explain why this information doesn’t apply to your submission:

(For large datasets, or papers with a very large number of statistical tests, you may upload a single table file with tests, Ns, etc., with reference to page numbers in the manuscript.)

**Additional data files (“source data”)**

- We encourage you to upload relevant additional data files, such as numerical data that are represented as a graph in a figure, or as a summary table
- Where provided, these should be in the most useful format, and they can be uploaded as “Source data” files linked to a main figure or table
- Include model definition files including the full list of parameters used
- Include code used for data analysis (e.g., R, MatLab)
- Avoid stating that data files are “available upon request”

Please indicate the figures or tables for which source data files have been provided:

We have provided source data files and MatLab scripts for Figure 3.

pSmad1/5/8 analysis: (pSMAD158_resultsfromMATLABcode. xlxs, pSmad158_MZgdf3Mut.m, pSmad158_WT.m)

analysis of eve1 expansion: (eve1_Insitu_quantification. xlxs, eve1_insitu_quantification.m)
